# Clinic of Prof. T. W. Brophy at the Chicago College of Dental Surgery

**Published:** 1885-05

**Authors:** H. L. Barnum


### CLINIC OF PROF. T. W. BROPHY AT THE CHICAGO COLLEGE OF DENTAL SURGERY.

REPORTED BY H. L. BARNUM, M. D.

CASE I.—Miss J. A., thirteen years of age, slight build. History—About eight months ago the left upper central incisor became very sensitive, without apparent decay or discoloration. In three weeks an abscess formed and discharged. The tooth was drilled open, and the root treated and filled by a dentist in this city. In two weeks the discharge recurred from the former abscess. Came to me August 1, 1884. An examination revealed carious bone and the exposed end of the root. It was treated with aromatic sulphuric acid, but unsuccessfully. Finally an operation was decided upon. The patient was etherized, and the exposed end of the root with the carious bone removed. The wound and cavity were dressed antiseptically, and were kept open by means of a wax plug, to allow granulation to commence from the bottom. For dressings, boracic acid was used, because of its stimulating qualities.

Case II.—Mrs. F. H——; age twenty; American; family history good. In June last the patient was eating ice cream, when the spoon struck the gum accidentally. The pain was severe at the time, but she thought nothing of it. In a short time the tooth loosened, and an enlargement appeared over the root of the lower left incisor. It grew rapidly for a time, then ceased to become larger, and has increased but little since. Examination shows a tumor extending from the right central incisor to the left cuspid. The tumor is somewhat erectile, and has a cartilaginous appearance. Treatment—The tooth was removed and the parts dried, and kept so by means of bibulous paper, and a few drops of a four per cent. solution of hydrochlorate of cocaine applied to the surface of the tumor. In eight minutes free incisions were made, and the tumor removed without pain. The cavity was dressed antiseptically with boracic acid, using a pledget of cotton to fill the space. The wound was dressed every day for five days, when the patient was discharged.

Case III.—N. N., age four; previous health good. Present condition—A sinus opening on the chin over the mental process of the lower maxilla. History.—The trouble commenced two years ago, with alveolar abscess, the result of a fall, the abscess opening at the root of the right lower central incisor, and discharging for some time. It then ceased. A year ago a sore appeared on the chin, which could not be healed. Some carious bone was removed and the trouble ceased for a time, only to renew again. Examination shows the death of the right lower central incisor, and caries of the mental process of the maxilla, dependent on chronic alveolar abscess. At the first sitting the pulp canal was opened from the lingual surface, and a solution of carbolic acid forced through the opening, making its way out at the sinus. The root was filled with liquid gutta-percha. A week later the patient was etherized, and an incision made on the labial side. The carious bone was scraped away, and the wound dressed with boracic acid and cotton. The dressing was renewed every day for a week, when the patient was discharged cured.

